# Knockdown of a JmjC domain-containing gene *JMJ524* confers altered gibberellin responses by transcriptional regulation of GRAS protein lacking the DELLA domain genes in tomato

**DOI:** 10.1093/jxb/eru493

**Published:** 2015-02-13

**Authors:** Jinhua Li, Chuying Yu, Hua Wu, Zhidan Luo, Bo Ouyang, Long Cui, Junhong Zhang, Zhibiao Ye

**Affiliations:** ^1^Key Laboratory of Horticultural Plant Biology (MOE), Huazhong Agricultural University, Wuhan 430070, China; ^2^Key Laboratory of Horticulture Science for Southern Mountainous Regions, Ministry of Education; College of Horticulture and Landscape Architecture, Southwest University, Chongqing 400715, P. R. China

**Keywords:** DELLA, dwarfism, gibberellin, GRAS, JmjC, tomato.

## Abstract

Plants integrate responses to independent hormonal and environmental signals to survive adversity. In particular, the phytohormone gibberellin (GA) regulates a variety of developmental processes and stress responses. In this study, the Jumonji-C (JmjC) domain-containing gene *JMJ524* was characterized in tomato. *JMJ524* responded to circadian rhythms and was upregulated by GA treatment. Knockdown of *JMJ524* by RNAi caused a GA-insensitive dwarf phenotype with shrunken leaves and shortened internodes. However, in these transgenic plants, higher levels of endogenous GAs were detected. A genome-wide gene expression analysis by RNA-seq indicated that the expression levels of two *DELLA-like* genes, *SlGLD1* (‘*G*RAS protein *L*acking the *D*ELLA domain’) and *SlGLD2*, were increased in *JMJ524*-RNAi transgenic plants. Nevertheless, only the overexpression of *SlGLD1* in tomato resulted in a GA-insensitive dwarf phenotype, suggesting that *SlGLD1* acts as a repressor of GA signalling. This study proposes that *JMJ524* is required for stem elongation by altering GA responses, at least partially by regulating *SlGLD1*.

## Highlight

Jumonji-C (JmjC) domain-containing protein JMJ524 plays important roles in tomato growth and development through conferring altered gibberellin responses by transcriptional regulation of a GRAS protein lacking the DELLA domain gene *SGLD1*. These results broaden our understanding of the JmjC domain-containing proteins, which not only control genic DNA methylation but are also involved in the GA response.

## Introduction

Phytohormone gibberellins (GAs) are fundamentally involved in various aspects of plant growth and development, including internode elongation (Hedden and Kamiya, 1997; Xiao *et al.*, 2006). GA exists in nature in many different isoforms, but only a few of them can actively regulate plant growth. The bioactive isoforms of GA include GA_1_, GA_3_, GA_4_, and GA_7_, in which GA_12_ and GA_53_ are the main precursors converted to various GA intermediates (e.g. GA_9_ and GA_20_) and bioactive GAs (e.g. GA_1_ and GA_4_) by GA 20-oxidases (GA20ox) and GA 3-oxidases (GA3ox), respectively. GA3ox catalyses the final step in the synthesis of bioactive GAs, whereas GA 2-oxidase (GA2ox) plays a major role in converting active GAs (GA_1_ and GA_4_) and their precursors (GA_9_ and GA_20_) to inactive forms (Sun, 2011; Raddatz *et al.*, 2013). Bioactive GAs can trigger a plant GA response in which DELLA proteins play a major role. With bioactive GAs, the GA receptor GID1 can bind GA in the nucleus and/or cytoplasm (Sun, 2010). If the binding occurs in the cytoplasm, the GID1-GA complex induces traffic to the nucleus, binding the nuclear-localized DELLA proteins. Once the DELLA protein binds to the GID1-GA complex, ubiquitin E3 ligase complex SCF recognizes and ubiquitinates the former, which is then degraded by the 26S proteasome, subsequently triggering GA responses (Achard and Genschik, 2009; Harberd *et al.*, 2009).

DELLA proteins belong to GRAS (*G*AI, *R*G*A,* and *S*CARECROW), a subfamily of the plant-specific regulatory protein family. These proteins have important roles in exceedingly diverse processes, such as signal transduction and meristem maintenance and development (Bolle, 2004). Other than the conserved N- and C-terminal domains of all GRAS family members, DELLA proteins also contain a unique DELLA domain that is essential in its own degradation (Dill *et al.*, 2001). *Arabidopsis* contains five DELLA proteins: RGA, GAI, RGA-LIKE1 (RGL1), RGL2, and RGL3. These display overlapping and distinct functions in repressing GA responses (Sun, 2011). However, only a single DELLA protein, Procera (pro) and SLENDER1 (SLR1), has been identified in tomato and rice, respectively (Ikeda *et al.*, 2001; Bassel *et al.*, 2004; Bassel *et al.*, 2008). Loss-of-function mutation in *pro* can result in tomato displaying a GA-constitutive response phenotype, in which the mutant is taller and has non-serrated leaves, which can be resemble the wild type (WT) plants if treated with GA_3_ (Carrera *et al.*, 2012). The loss-of-function mutant of the *SLR1* gene also results in a constitutive GA response and exhibits rapid extension growth in seedling and sterile phenotypes. In particular, the truncation of DELLA in the *SLR1* gene produces a dwarf phenotype in rice (Ikeda *et al.*, 2001). As a repressor of GA signalling, the rice genome contains two *SLR1-LIKE* (*SLRL*) genes encoding proteins that have insufficient DELLA domain and function, whereas the overexpression of *SLRL*1 in transgenic rice inhibits shoot growth with a weaker effect compared with *SLR1* (Itoh *et al.*, 2005).

Jumonji-C (JmjC) proteins play important roles in plant growth and development (Chen *et al.*, 2011*b*). The JmjC domain was first defined based on the amino acid similarities of Jumonji (Jmj) proteins identified in a gene-trap screen for neural tube formation factors in mouse (Takeuchi *et al.*, 1995). These similarities were later suggested to regulate chromatin remodelling (Clissold and Ponting, 2001). JmjC domain-containing proteins were initially predicted to be involved in demethylation of modified arginine or lysine amine groups within histones (Trewick *et al.*, 2005; Tsukada *et al.*, 2006). This proposition was confirmed using a technique based on biochemical purification, which identified the JmjC domain-containing protein JHDM1, an H3K36-specific demethylase (Tsukada *et al.*, 2006). Human Jmj-type enzymes are involved in various pathological processes, including development, cancer, inflammation, and metabolic diseases (Johansson *et al.*, 2014). For example, Jarid2/Jumonji, a member of the Jmj factor family, regulates cardiovascular development (Mysliwiec *et al.*, 2012). Lung cancer-associated JmjC domain protein MDIG is a common feature of non-small cell lung cancer (Lu *et al.*, 2009). Inhibition of a JmjC domain-containing histone demethylase 1B (JHDM1B) contributes to acute myeloid leukemia cell proliferation (Nakamura *et al.*, 2013). Moreover, the H3K27-specific JMJs (KDM6 subfamily members JMJD3 and UTX) regulate disease-relevant inflammatory responses (Kruidenier *et al.*, 2012). *JMJD5* is a participant in both human and *Arabidopsis* circadian systems (Jones *et al.*, 2010). *Arabidopsis JMJ30* also standardizes the circadian clock and period length (Lu *et al.*, 2011).

Many JmjC domain-containing proteins have recently been identified in plants (Lu *et al.*, 2008; Zhou and Ma, 2008), but only a few of them have been functionally characterized. The first plant JmjC genes characterized were *Early Flowering 6* (*ELF6*) and *Relative of Early Flowering 6* (*REF6*) from *Arabidopsis*. ELF6 acts as floral repressor, whereas REF6 functions as floral activator (Noh *et al.*, 2004). Subsequently, more functions of different JmjC domain-containing proteins from *Arabidopsis* and rice were distinguished. In *Arabidopsis*, the improved expression of ‘increase in bonsai methylation 1’ (IBM1), another JmjC domain-containing protein, represses genic DNA cytosine methylation, possibly via demethylation at H3K9 (Saze *et al.*, 2008), and it has also been confirmed to protect the transcribed genes, but not the transposons, from DNA methylation at CHG sites (Miura *et al.*, 2009). *JMJ706*, a rice homologue of IBM1, encodes an H3K9 demethylase and participates in the regulation of floral organ formation (Sun and Zhou, 2008). Meanwhile, rice JmjC domain-containing protein JMJ703 is a histone lysine demethylase that is required for stem elongation by regulating the expression of cytokinin oxidase genes (Chen *et al.*, 2013). Posttranscriptional gene silencing has been reported in *Arabidopsis* due to mutations in the Jmjc domain protein H3K4me2/3 demethylase JMJ14 (Le Masson *et al.*, 2012).

Using an oligonucleotide microarray, Gong *et al.* (2010) previously investigated drought stress and identified a JmjC domain-containing gene, *JMJ524*, from tomato with different expression profiles in drought-tolerant introgression lines (ILs) and their recurrent parent M82. In this study, *JMJ524* was further characterized and shown to respond to circadian rhythms and GA treatment. The knockdown of *JMJ524* caused a severe GA-insensitive dwarf phenotype. The results of RNA-seq transcriptome analysis indicated that two *Procera* homologues, namely *SlGLD1* (*G*RAS protein *L*acking the *D*ELLA domain) and *SlGLD2*, were upregulated in *JMJ524*-RNAi transgenic plants. Transgenic tomato plants overexpressing *SlGLD1* also exhibited a GA-insensitive dwarf phenotype. These findings demonstrate that in tomato, *JMJ524* might be required for stem elongation through the regulation of *SlGLD1* expression.

## Materials and methods

### Plant materials and growth conditions

Tomato (*Solanum pennellii* LA0716 and *S. lycopersicum* cv. M82) plants were grown in a glasshouse with natural illumination (day/night temperature 25/18°C; relative humidity ~55%). For gene expression analysis, six-week-old seedlings were treated with 100 μM GA_3_ or a mock to detect the gene response to circadian rhythm and GA. The leaves were then collected at designated time points, immediately frozen in liquid nitrogen, and stored at –80°C until use. A total of 100 µM GA_3_ solution containing 0.02% Tween 20 was sprayed on the 30-d-old seedlings with an interval of 3 d for 2 weeks to determine the sensitivity of dwarf phenotypes.

### Gene isolation and tomato transformation

In previous studies on drought stress in tomato ILs, differential transcript expression profiles were observed in the drought-tolerant ILs and M82 for the *JMJ524* gene by using an oligonucleotide microarray (Gong *et al.*, 2010). The tomato *JMJ524* coding sequence (CDS) was amplified using the polymerase chain reaction (PCR) from *S. pennellii* cDNA using gene-specific primers (GSPs: *JMJ524*-OE) (Supplementary Table S1) based on a unigene sequence (SGN-U576053, http://solgenomics.net/). The reverse transcription PCR (RT-PCR) product was recovered, cloned into pMD 18-T vector (TaKaRa), and sequenced (BGI, China). Multiple alignment of homologous sequences of *JMJ524* from other species was conducted using ClustalW (http://www.ebi.ac.uk/clustalw/). A phylogenetic tree was constructed using the neighbour-joining method with MEGA (version 5.05) software.

The RNA interference vector was constructed by amplifying a 357bp fragment from *JMJ524* CDS using GSPs with 5′-attB1 and 5’-attB2 extensions on forward and reverse primers, respectively (*JMJ524*-Ri) (Supplementary Table S1; 5′-attB1 and 5′-attB2 extensions are underlined). A recombination reaction was performed between the PCR product and pHellsgate 2 vector (Invitrogen, USA) using BP clonase (Invitrogen, USA) according to the manufacturer’s instructions. For *SlGLD1* and *SlGLD2* overexpression, the binary plasmid vector pMV2 (Yang *et al.*, 2011), which carries spectinomycin resistance and neomycin phosphotransferase II genes for bacterial and transformed plant selections, respectively, was used. Meanwhile, the binary plasmid was formed by inserting *SlGLD1* or *SlGLD2* cDNA in a sense orientation into the vector after the cauliflower mosaic virus 35S promoter. All plasmids were transformed into the tomato cultivar M82 by *Agrobacterium tumefaciens* (strain C58) mediated transformation. After regenerated shoots were screened on selection medium containing kanamycin, the transgenic plants were further verified by PCR using genomic DNA as a template and the 35S promoter forward and gene-specific reverse primers.

### RNA isolation and real-time RT-PCR

Gene expression patterns were examined by isolating total RNA using the TRIzol reagent (Invitrogen, USA). DNase I-treated RNA was reverse transcribed using an M-MLV Reverse Transcription enzyme (Invitrogen, USA). The resulting cDNA was used for real-time RT-PCR. Real-time RT-PCR was performed with the SYBR Green I Master kit (Roche, Switzerland) using primers (Supplementary Table S1) specific for genes, with β-actin (SGN-U580609) transcripts as an internal control. PCR amplification consisted of an initial incubation at 95°C for 5min, followed by 40 cycles of 95°C for 10 s, 58°C for 15 s, and 72 °C for 20 s. Data were collected during extension and melting curve acquisitions; analyses were also performed in real time. PCR products were monitored with a LightCycler 480 (Roche, Switzerland) PCR system.

### Transcriptional activation analysis in yeast cells

For transactivation assays, the CDS of *JMJ524* was amplified with PCR using the generalized system of preferences (GSPs) (JMJ524-Y; Supplementary Table S1), which were designed to introduce *Sma*I and *Pst*I restriction sites (underlined). The PCR product was fused in frame to the yeast GAL4 DNA-binding domain of the pGBKT7 vector (Clontech, USA) after its digestion with *Sma*I and *Pst*I. pGBKT7-JMJ524 and pGBKT7 (negative control) were separately transformed into the yeast strain AH109 following the manufacturer’s instructions (Clontech, USA). The transformed strains were streaked on SD/–Trp or SD/–Ade/–His/–Trp medium supplemented with X-α-Gal (20mM) to assay another yeast reporter (*MEL1*) gene. The transactivation activity of each gene was evaluated according to their growth status.

### RNA-seq and functional assignment

Young leaves from 50-d-old WT and *JMJ524-*RNAi (Ri-14) plants were harvested, and total RNA was isolated using TRIzol reagent (Invitrogen, USA). At least 10 µg of total RNA per sample was enriched for mRNA by conducting purification using oligo(dT) magnetic beads. cDNA synthesis was performed by random hexamer priming after the mRNA was fragmented into 200bp fragments. Buffer, dNTPs, RNase H, and DNA polymerase I were added to synthesize the second strand. The double-stranded cDNA was purified with a QiaQuick PCR extraction kit and washed with EB buffer prior to end repair and single nucleotide A (adenine) addition. Finally, sequencing adaptors were ligated to the fragments, which were purified via agarose gel electrophoresis and enriched by PCR amplification. The products were submitted for single-end sequencing with a read length of 49bp using an Illumina HiSeq^TM^ 2000. Raw sequence data were then filtered to remove low-quality tags (tags with unknown nucleotide, ‘N’), empty tags (no tag sequence between the adaptors), and tags with only one copy (which might result from sequencing errors). For tag annotation, clean tags were mapped to the tomato transcriptome reference database ITAG2.3 (http://solgenomics.net/itag/release/2.3/list_files), and no more than two base mismatches were allowed in the alignment.

Differences in gene expression were compared by statistically analyzing the tag frequency in each RNA-seq tag library according to previously described methods (Audic and Claverie, 1997). False discovery rate (FDR) was used to determine the value of threshold *P* in multiple tests. An FDR ≤0.001 and an absolute value of the log_2_ ratio ≥1 were used as thresholds to determine significant differences in gene expression. The differentially expressed genes were used in KEGG pathway enrichment analyses. Tomato transcripts were annotated by performing a BLAST search against the non-redundant database at NCBI. The values of reads per kb of exon model per million mapped reads were used to evaluate the expressed value and to quantify transcript levels.

### Paraffin sectioning

All tissues from 50-d-old *JMJ524*-RNAi and WT seedlings were harvested and fixed in formalin/acetic acid/alcohol solution for 24h, stained with hematoxylin, dehydrated with an ethanol gradient series (15, 30, 50, and 70%), infiltrated with paraffin using chloroform as a solvent, and gradually embedded with paraffin. The specimens were sectioned (8–10 µm) using a Leica RM2245 (Germany) semi-motorized rotary microtome and subsequently mounted on microscope slides. Paraffin was removed from the specimens by immersing the slides in xylene (twice for 20min) and embedding them with neutral resin. The slides were then placed in an oven at 42°C until dried. Photomicrographs were taken using a Nikon ECLIPSE 80i microscope.

### Quantification of endogenous GAs

Tomato leaves (1g) were frozen in liquid nitrogen, finely ground, and then extracted with 15ml methanol containing 20% water (v/v) at 4°C for 12h. The following labelled GAs were added as internal standards before grinding: [^2^H_2_]GA_1_ (1.00ng g^–1^), [^2^H_2_]GA_3_ (1.00ng g^–1^), [^2^H_2_]GA_4_ (2.00ng g^–1^), [^2^H_2_]GA_12_ (2.00ng g^–1^), [^2^H_2_]GA_20_ (2.00ng g^–1^), and [^2^H_2_]GA_53_ (4.00ng g^–1^). Further sample preparation and analyses were performed as previously described (Chen *et al.*, 2011*a*; Li *et al.*, 2012).

### Analysis of cytosine methylation by HPLC

The cetyltrimethylammonium bromide (CTAB) plant DNA extraction method (Murray and Thompson, 1980) was modified to isolate tomato DNA. A 50 µl volume of 70% perchloric acid was added to 100 µl DNA solution (containing ~25 µg DNA) and was hydrolysed at 95°C for 50min. The pH was adjusted to a value of 3 to 5 with 1M KOH. The sample was subsequently spun at 12 000*g* for 5min; the resulting supernatant was used for high pressure liquid chromatography (HPLC) analysis. HPLC analysis was performed using a Waters 2695 separation module equipped with a Waters 2998 DAD detector and a ZORBAX-AQ C18 column (4.6×250mm, 5 μm particle size: Agilent, USA). Sample was detected at 280nm (UV) with an injection volume of 10 μl. The mobile phase comprised 0.1% aqueous formic acid as eluent A and methanol as eluent B with the following gradient: 5–30% eluent B over 30min with a flow rate of 1ml min^–1^. Cytosine (C) and methylcytosine (MC) contents were assessed via co-migration under the same HPLC conditions with commercial standards (Sigma). MC percentages were calculated with the following formula: %MC = (MC/(C + MC)) × 100.

## Results

### Isolation, transactivation activity assay, and expression pattern of *JMJ524*

**Fig. 1.**
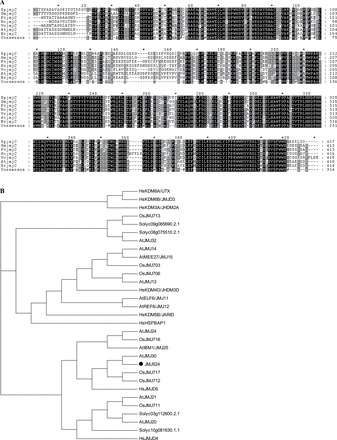
Comparison and phylogenetic analysis of amino acid sequences of plant *JMJ524* genes. (A) CLUSTAL alignment of deduced amino acid sequence of *JMJ524* and those of *Glycine max* (GmjmjC: I1LJT0), *Populus trichocarpa* (PtjmjC: B9GM97), *Ricinus communis* (RcjmjC: B9RI82), *Vitis vinifera* (VvjmjC: F6GU98), *Arabidopsis thaliana* (AtjmjC: Q9LT40), and *Brassica rapa* (BrjmjC: G1FE06). Black and grey boxes indicate identical and similar amino acids, respectively. (B) Phylogenetic analysis of JMJ524 (indicated by a black circle) and JmjC proteins from other species (species and GenBank accession numbers are shown in Supplementary List S1), including proteins from human, *Arabidopsis thaliana*, and *Oryza sativa* indicated by Hs, At, and Os prefixes, respectively. The phylogenetic tree was generated by ClustalW2 using standard parameters of the neighbour-joining method in MEGA (version 5.05).

In previous studies on drought stress in tomato ILs, *JmjC524* (SGN-U218046) was identified as a differentially expressed gene in comparison with drought-tolerant ILs and M82 (Gong *et al.*, 2010). Using RT-PCR, the full-length (1254bp) open reading frame of *JMJ524* from the wild tomato species *S. pennellii* LA0716 was isolated and cloned. *In silico* analysis illustrated that the *JMJ524* genomic DNA sequence contains five introns. According to the SGN (http://solgenomics.net) genome annotation database, four other JmjC domain-containing proteins, namely Solyc03g112600.2.1, Solyc09g065690.2.1, Solyc10g081630.1.1, and Solyc08g075510.2.1, were identified in tomato，but these proteins share low similarity with *JMJ524*. Using NCBI pBLAST and *JMJ524* amino acid sequence, the sequences of the most homologous proteins were retrieved from other species in the database. A CLUSTAL alignment of all sequences indicated high conservation among the JmjC domain-containing proteins (Fig. 1A). The phylogenetic tree constructed based on the amino acid sequences of JMJ524 and JmjC proteins from other representative organisms (*Arabidopsis thaliana*, human, and rice) showed that JMJ524 was significantly correlated with its counterpart *Arabidopsis* AtJMJ30 protein, whereas it was most distant from human HsKDM6A protein (Fig. 1B).

**Fig. 2.**
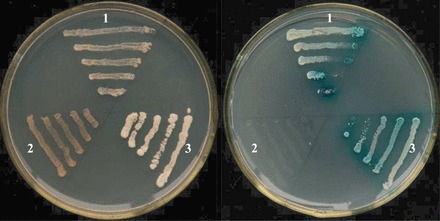
Transcriptional activation of JMJ524 in yeast. JMJ524 and GAL4 DNA-binding domain fusion protein were expressed in yeast strain AH109 (1). Vectors pGBKT7 and pGBKT7-53+pGADT7-T were expressed in yeast as negative (2) and positive controls (3), respectively. Yeast streaks were cultured on SD/–Trp and SD/–Ade/–Leu/–Trp media containing X-α-GAL for assaying another yeast reporter LacZ.

JmjC domain-containing proteins have been identified in numerous eukaryotic proteins, including PHD, C2H2, ARID/BRIGHT, and zinc fingers, which contain domains typical of transcription factors (Clissold and Ponting, 2001; Elkins *et al.*, 2003). Transactivation activity is a defining feature for a transcription factor. To identify whether JMJ524 functions as a transcriptional activator the yeast two-hybrid analysis was used. To this end, a GAL4 DNA-binding domain JMJ524 fusion protein was expressed in yeast cells that were subsequently assayed for their ability to activate transcription from the GAL4 sequence. Yeast cells carrying either control (pGBKT7) or fusion plasmid grew well on SD/–Trp medium, indicating that the analysis system was reliable. However, on the SD/–Ade/–His/–Trp medium, the cells transformed with the control plasmid could not grow, whereas those transformed with fusion plasmid grew normally (Fig. 2). Moreover, JMJ524 promoted yeast growth in the absence of histidine and adenine and exhibited X-α-gal activity. The vector control pGBKT7 did not achieve the same result (Fig. 2). These data confirmed that *JMJ524* functions as a transcriptional activator in yeast and probably acts as a transcription factor in tomato.

**Fig. 3.**
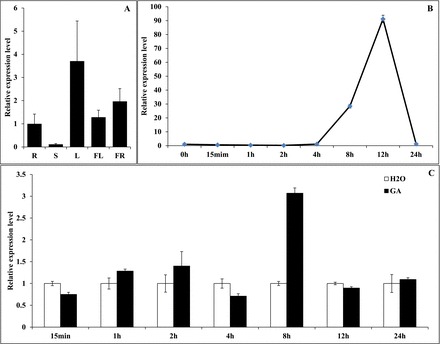
Analysis of *JMJ524* expression in tomato. (A) Real-time RT-PCR analysis of *JMJ524* expression in different tissues (R, root; S, stem; L, leaf; FL, flower; FR, fruit) of *S. pennellii* LA0716. (B) The *JMJ524* transcript level under circadian rhythm in *S. pennellii* LA0716 plants. (C) Real-time RT-PCR analysis of *JMJ524* expression in response to GA treatment. Expression of GA-treated *S. pennellii* LA0716 plants was compared with that in untreated plants after normalization of values with reference to the tomato β-actin gene and is presented as the relative expression level. All samples were collected at the time points indicated (‘h’ refers to hours after treatment) from three biological replicates of each treatment. Error bars indicate SE of three replicates.

The expression level of *JMJ524* showed obvious tissue/organ specificity in tomato’s wild relative *S. pennellii*, and it was higher in the leaves but lower in stem tissue (Fig. 3A). Previous studies have shown that the expression of JMJ30 (Lu *et al.*, 2011) and JMJD5 (Jones *et al.*, 2010) exhibit circadian regulation. In order to make clear that their counterpart JMJ524 acts in a similar way, the expression profile of *JMJ524* in response to circadian rhythms was assessed using real-time RT-PCR. The results showed that the expression of *JMJ524* was quickly inhibited and then began to accumulate after 4h, continued to increase until it reached the highest level at 12h, >90-fold higher than the initial level, but returned to the 0h level 24h later (Fig. 3B). Meanwhile, *JMJ524* expression was also evaluated in leaves from plants that had been treated with GA_3_ to determine whether it is regulated by this growth regulator. In order to eliminate the influence of the circadian rhythm, the transcription levels of *JMJ524* in GA_3_-treated and untreated plants at the same time point were compared. The result of the investigation revealed that the transcript levels of *JMJ524* substantially increased at 8h after GA treatment (Fig. 3C). These expression patterns indicate that *JMJ524* was regulated by GA_3_ and the circadian rhythm.

### Suppression of tomato *JMJ524* caused a GA-insensitive dwarf phenotype

**Fig. 4.**
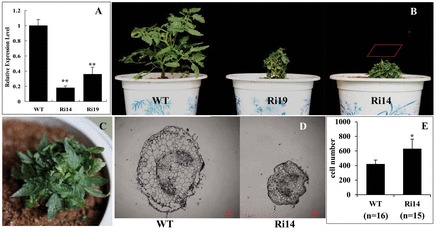
Knockdown of *JMJ524* causes a severe dwarfism phenotype. (A) Expression of *JMJ524* in transgenic and WT plants was examined in the fifth leaf through real-time PCR analyses. Double asterisks (**, *P* < 0.01) denote statistically significant differences between transgenic and WT lines. (B) Phenotype of plants (about three months old) in *JMJ524*-RNAi (Ri) and WT lines with magnified view in the box (C). (D) Phenotype of transverse sections of stems collected from Ri14 (left) and WT (right) plants showing epidermal cells. Scale bar, 100 μm. (E) Comparison of stem epidermal cell lengths between WT and mutant plants. Single asterisk (*, *P* < 0.05) denotes statistically significant differences between transgenic and WT lines. This figure is available in colour at *JXB* online.

**Fig. 5.**
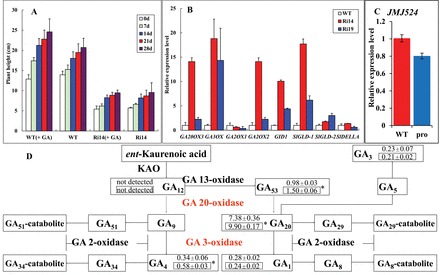
Increased GA level and expression of GA biosynthetic and responsive genes in *JMJ524*-RNAi lines. (A) Plant height is displayed as the average of eight plants. Error bars show SEs by spraying GA_3_ at 3 d intervals starting from 30 d after inoculation. (B) Expression of tomato GA biosynthetic and responsive genes in *JMJ524-*RNAi lines (Ri14 and Ri19) and WT. (C) Expression of JMJ524 in wild type (WT) and *procera* (*pro*) mutant plants as revealed by real-time RT-PCR analysis. (D) GA biosynthesis pathway. GA 20-oxidase and GA 3-oxidase both have significantly upregulated expression (*P* < 0.05). Numbers indicate endogenous GA levels in WT (upper) and transgenic plants (lower, boxed). *, *P* < 0.05. Data (ng g^–1^ fresh weight) is presented as mean ± SE from three technical replicates using two transgenic lines. This figure is available in colour at *JXB* online.

*JMJ524* was functionally characterized by generating transgenic tomato lines with overexpression or RNAi silencing of *JMJ524*. The expression level of the *JMJ524* gene in transgenic (T_0_ and T_1_) and control plants was examined at the five-leaf stage by conducting real-time PCR analyses. Two significantly downregulated *JMJ524* T_1_ transgenic lines, Ri-14 and Ri-19 (Fig. 4A), were selected for further analysis. The transgenic and WT plants were kept under the same conditions, and plant height was measured 50 d after germination. The *JMJ524*-RNAi plants showed a severe dwarf phenotype (Supplementary Figure S1A), whereas no obvious change of morphology was observed in plants overexpressing *JMJ524* (data not shown). The average height of the Ri-14 line was 5.7cm, which was 73% less than that of the control at 21.1cm. Moreover, the leaves of the transgenic plants were smaller (Supplementary Figures S1B, C). The dwarf phenotype was more severe toward the reproductive stage, with shortened stems and wizened leaves (Figs 4B, C). Meanwhile, results of the histological analysis of the stem cross-section cell (Fig. 4D) revealed that cell size was smaller in the *JMJ524*-RNAi lines, which contain more cells in their stems (Fig. 4E), implying a reduction in cell elongation in the dwarf phenotype. In plants, the dwarf phenotype is probably caused by GA deficiency. Accordingly, endogenous GA content in *JMJ524*-RNAi transgenic lines was investigated. As shown in the schematic representation of GA biosynthesis (Fig. 5D), the endogenous levels of three GAs (GA53, GA20, and GA4) in *JMJ524*-RNAi plants were significantly higher than those in the WT. High concentration of GAs could enhance plant stem elongation and induce a tall plant phenotype. These adverse results signified that the dwarf phenotype of *JMJ524*-RNAi transgenic plants was due to other reasons, not GA deficiency.

### *JMJ524* regulates the expression of GA biosynthesis and response genes

The molecular mechanism of the *JMJ524*-mediated dwarf plant phenotype was further examined and the molecular role of *JMJ524* was studied by performing a comparative analysis of the *JMJ524*-RNAi and WT leaf transcriptomes (RNA-seq). Most of the RNA-seq data parameters were comparable between the two samples (Supplementary Table S2). For the WT, up to 12 503 639 clean reads, which corresponded to a total length of 612 678 311bp, were sequenced and accounted for 89% of the tomato transcriptome reference database ITAG2.3. Similar to the WT (Supplementary Table S2), up to 11 898 385 clean reads, which corresponded to a total length of 583 020 865bp, were obtained from the *JMJ524*-RNAi transgenic plants, and these accounted for 88% of the tomato transcriptome reference database ITAG2.3. These results suggest that RNA-seq transcriptome covers most of the reference genome and most of the expressed genes were detected.

The putative differentially expressed genes comparing *JMJ524*-RNAi and WT plants were identified by applying FDR ≤0.001 and a log_2_ ratio ≥1 as thresholds to determine significance levels. Using these criteria, 3026 genes were identified as being upregulated in the *JMJ524*-RNAi plant compared with the WT. Meanwhile, transcripts corresponding to 293 genes decreased by more than 2-fold in the *JMJ524*-RNAi plant (Supplementary File S1), revealing that a far greater number of genes are upregulated when *JMJ524* expression is suppressed. The result of KEGG pathway enrichment analyses using differentially expressed genes indicated that the absence of *JMJ524* expression altered GA metabolism because the expression levels of GA biosynthesis genes, including GA20oxs and GA3oxs, were significantly greater in the *JMJ524*-RNAi plants than in WT, as confirmed by real-time PCR (Fig. 5B). The upregulated expression of GA biosynthesis genes in RNAi lines was consistent with the increased endogenous GA levels in *JMJ524*-RNAi transgenic plants.

A high concentration of GAs could not explain the dwarf phenotype of *JMJ524*-RNAi transgenic lines; however, besides GA biosynthesis, the top differentially expressed genes also included several GA response-related genes (Table 1). Considering that the dwarf phenotype associated with GA insensitivity can also be obtained by blocking the GA-GID1-DELLA signalling pathway (Sun, 2011) and that silencing of *SlDELLA* in tomato can produce a taller plant with decreased expression of GA20ox and GA3ox (Marti *et al.*, 2007), we suspected that the dwarf phenotype of *JMJ524*-RNAi might be associated with a GA response. Thus, a GA spraying assay was used to evaluate the GA sensitivity of the *JMJ524*-RNAi dwarf plants. After three weeks of GA spraying, the dwarf plants could not be rescued (Fig. 5A), indicating that the GA response was blocked and that the *JMJ524*-RNAi dwarf plants were GA insensitive. RNA-seq and real-time PCR results showed that the differentially expressed genes associated with the GA response did not contain the *SlDELLA* gene in the *JMJ524*-RNAi dwarf plants (Fig. 5B). However, the expression of two genes encoding *G*RAS protein *L*acking the *D*ELLA domain proteins (*SlGLD1*, Solyc10g086380.1.1; and *SlGLD2*, Solyc10g086370.1.1) was significantly upregulated (Fig. 5B). Previous research has confirmed that overexpression of *SLRL*1 in transgenic rice inhibits shoot growth (Itoh *et al.*, 2005); thus, we considered that *JMJ524* might regulate stem development and a GA response by regulating *SlGLD1* or *SlGLD2,* but not *SlDELLA.*

**Table 1.** Genes involved in GA biosynthesis and signalling response

Gene ID (SGN)	Length (bp)	log_2_ ratio	Homology / organism / accession number	E-value
Solyc02g062490.2.1	763	9.06	Gibberellin 20 oxidase / *Ricinus communis* / XP_002517541.1	2.74e-43
Solyc11g072310.1.1*	1140	8.80	Gibberellin 20-oxidase-3 / *Solanum lycopersicum* / AAD15756.1	0
Solyc04g008670.1.1	1074	8.15	Gibberellin 20-oxidase / *Ricinus communis* / XP_002518816.1	3.70e-129
Solyc03g006880.2.1	1468	0.93	Gibberellin 20-oxidase-1 / *Solanum lycopersicum* / AAD15755.1	0
Solyc06g035530.2.1	1358	0.56	Gibberellin 20-oxidase-2 / *Solanum lycopersicum* / AAD15754.1	0
Solyc01g093980.2.1	1448	0.07	Gibberellin 20-oxidase 4 / *Solanum lycopersicum* / ACC86835.1	0
Solyc01g058250.1.1*	1047	8.19	Gibberellin 3-oxidase / *Populus trichocarpa* / XP_002324270.1	6.50e-107
Solyc10g007570.2.1	808	7.97	Gibberellin 2-oxidase 3 / *Nicotiana tabacum* / ABO70985.1	1.10e-93
Solyc02g070430.2.1	1281	5.53	Gibberellin 2-oxidase 1 / *Solanum tuberosum* / ABS19663.1	0
Solyc07g056670.2.1*	1209	3.46	Gibberellin 2-oxidase 2 / *Solanum lycopersicum* / ABK15560.1	0
Solyc07g061720.2.1	1227	3.24	Gibberellin 2-oxidase / *Solanum lycopersicum* / ABO27635.1	0
Solyc01g079200.2.1	1155	2.65	Gibberellin 2-oxidase / *Solanum lycopersicum* / ABO27634.1	0
Solyc07g061730.2.1	1299	0.81	Gibberellin 2-oxidase / *Solanum lycopersicum* / ABO27636.1	0
Solyc10g005360.2.1	940	0.07	Gibberellin 2-oxidase 1 / *Nicotiana sylvestris* / gb|AAO92303.1	1.11e-98
Solyc01g098390.2.1	1138	-7.41	Gibberellin 2-oxidase / *Solanum lycopersicum* / ABO27632.1|	0
Solyc01g058040.1.1	369	-1.51	Gibberellin 2-oxidase / *Solanum lycopersicum* / ABO27632.1	3.60e-38
Solyc02g080120.1.1*	1143	-0.85	Gibberellin 2-oxidase 1 / *Nicotiana sylvestris* / AAO92303.1	2.64e-180
Solyc01g058030.1.1	654	-0.19	Gibberellin 2-oxidase / *Solanum lycopersicum* / ABO27632.1	9.853e-70
Solyc09g074270.2.1	1576	3.15	GID1-like gibberellin receptor / *Solanum lycopersicum* / CAP64330.1	0
Solyc06g008870.2.1*	1666	2.72	GID1-like gibberellin receptor / *Solanum lycopersicum* / CAP64330.1	5.79e-169
Solyc01g098390.2.1	1782	1.07	Gibberellin receptor GID1 / *Ricinus communis* / XP_002512310.1	1.37e-155
Solyc01g059950.1.1	843	8.91	DELLA protein RGL1 / *Ricinus communis* / XP_002519213.1	4.70e-42
Solyc12g099220.1.1	1728	5.88	DELLA protein GAI1 / *Ricinus communis* / XP_002523464.1	2.20e-110
Solyc10g086380.1.1*	1542	2.81	DELLA protein GAI / *Solanum lycopersicum* / Q7Y1B6.1	1.27e-130
Solyc10g086370.1.1*	1533	0.53	DELLA protein GAI / *Solanum lycopersicum* / Q7Y1B6.1	4.54e-96
Solyc11g011260.1.1*	1767	-0.08	DELLA protein GAI / *Solanum lycopersicum* / Q7Y1B6.1	0

Data according to previous study by RNA-seq analysis in *JMJ524*-RNAi (Ri14) and WT. *, genes selected for qRT-PCR.

Given that the stem cell number of *JMJ524*-RNAi lines was significantly larger than that of the WT (Fig. 4E), we also comprehensively compared the expression of cell cycle-related genes in the WT and *JMJ524*-RNAi transgenic plants using the RNA-seq transcriptome data. Four cell cycle-related genes (Solyc10g074720.1.1, Solyc12g088530.1.1, Solyc05g051410.2.1, and Solyc02g092980.2.1) were significantly and differentially expressed in the WT and *JMJ524*-RNAi plants with an increased expression in the *JMJ524*-RNAi lines (Supplementary Table S3), as confirmed through real-time PCR (data not shown). These results showed that the abnormal expression patterns of the cell cycle-related genes might be related to the abnormal stem elongation of the *JMJ524*-RNAi transgenic plants. The expression of these cell cycle-related genes might be indirectly regulated by a GA response.

### Overexpression of *SlGLD1* in tomato caused a dwarf phenotype

**Fig. 6.**
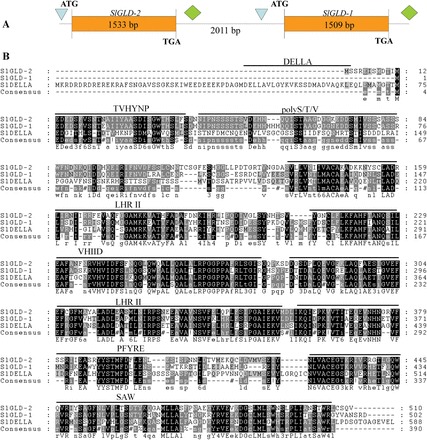

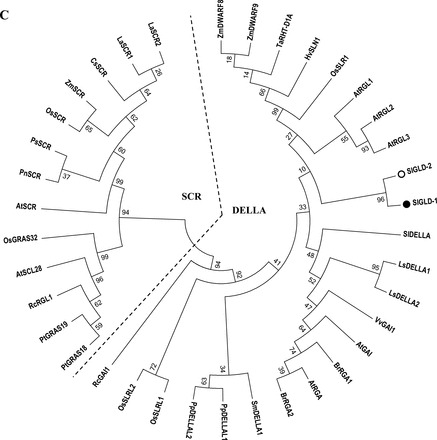
Schematic representation and comparison of amino acid sequences of SlDELLA, SlGLD1, and SlGLD2, as well as phylogenetic analysis of SlGLDs in the GRAS family. (A) Schematic representation of *SlGLD1* and *SlGLD2* genes. Boxes represent exons; triangles and diamonds represent positions of transcription start and poly(A) sites, respectively. (B) Comparison of amino acid sequences of SlDELLA, SlGLD1, and SlGLD2. Black and grey boxes indicate identical and similar amino acids, respectively. Lines above the alignment indicate locations of conserved regions in GRAS proteins as defined by Pysh *et al.* (1999). (C) Phylogenetic analysis of SlGLD1, SlGLD2, and other GRAS proteins from other species (species and GenBank accession numbers are shown in Supplementary List S2) generated using the VHIID region. SlGLD1 is indicated by a black circle. The phylogenetic tree was constructed using the MEGA5 program with the neighbour-joining method using 1000 bootstrap replicates. This figure is available in colour at *JXB* online.

*SlGLD1* and *SlGLD2* are present in tandem in the tomato genome (Fig. 6A). The full-length ORFs of *SlGLD1* and *SlGLD2* were 1509 and 1533bp, respectively. SlGLD1 shared 66% identity with SlGLD2 and 50% identity with SlDELLA, whereas SlGLD2 and SlDELLA were 45% identical at the amino acid sequence level. The C-terminal conserved domains of the SlGLDs, such as VHIID, leucine heptad repeat II, PFYRE, and SAW, showed high similarity with SlDELLA (procera) protein. Interestingly, although the N-terminal regions of the SlGLDs contained the conserved domains of DELLA proteins, such as TVHYNP and Ser/Thr/Val-rich (polyS/T/V), the DELLA domains were deleted (Fig. 6B). Phylogenetic analysis of the SlGLDs and other GRAS proteins in plants using the VHIID domain showed that both SlGLD1 and SlGLD2 are categorized under the DELLA group, suggesting that they may function in a GA response similar to the Procera protein.

**Fig. 7.**
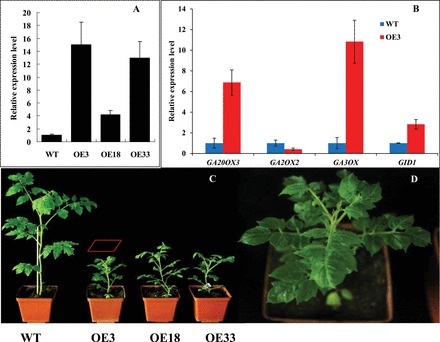
Functional analysis of the *SlGLD1* in tomato. (A) Overexpression of *SlGLD1* in transgenic tomato plants. Expression levels of *SlGLD1* in transgenic and WT control plants at the five-leaf stage were examined using real-time PCR analyses. (B) Expression of genes involved in GA biosynthesis and response in the *SlGLD1* overexpression line (OE3) and WT. (C) Developmental phenotype of 50-d-old *SlGLD1*-overexpressed plants (left) with magnified view to the far right representing the plant with the box (D). This figure is available in colour at *JXB* online.

To investigate whether SlGLD1 and SlGLD2 function in a GA response, transgenic plants with overexpressed *SlGLD1* or *SlGLD2* genes were generated. A total of 35 and 26 independent transgenic lines of *SlGLD1* and *SlGLD2* were obtained, respectively. No visible alteration in phenotype was observed in *SlGLD2*-overexpressed transgenic plants, but eight of the 35 *SlGLD1*-overexpressed transgenic plants showed an obvious dwarfism phenotype similar to the *JMJ524*-RNAi transgenic lines (Figs 7C, D). Three independent transgenic lines, namely OE3, OE18, and OE33, with significantly increased *SlGLD1* expression and obvious an dwarfism phenotype, were selected for further study (Fig. 7A). Gene expression analysis indicated that GA biosynthesis-related genes (GA20oxs and GA3oxs) and the GA response-related gene (*GID1*) were significantly upregulated in *SlGLD1*-overexpressed transgenic lines (Fig. 7B). Their expression patterns were similar to those in the *JMJ524*-RNAi transgenic plants. These results implied that *JMJ524* might partially affect stem elongation and a GA response by regulating the expression of *SlGLD1*.

### *JMJ524*-RNAi plants have increased DNA methylation

**Fig. 8.**
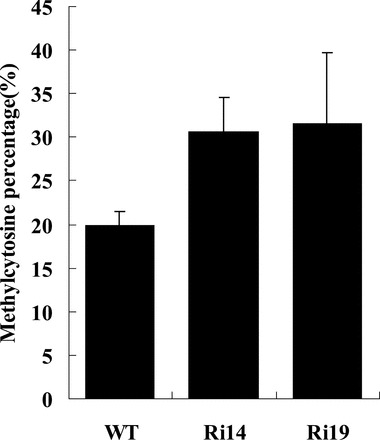
Total percentage of methylcytosine in *JMJ524*-RNAi lines (Ri14 and Ri19) and the WT. Data are presented as mean ± SD from three technical replicates using two independent genomic DNA sets prepared from 50-d-old seedlings.

It has been reported that JmjC domain-containing proteins play important roles in DNA demethylation (Saze *et al.*, 2008), so the global DNA methylation level of *JMJ524*-RNAi transgenic plants was evaluated by HPLC. The overall cytosine methylation levels of tomato genomic DNA prepared from the young upper leaves of *JMJ524*-RNAi and WT plants were measured and the 5^m^C content in *JMJ524*-RNAi genomic DNA was shown to be significantly higher than in the WT (Fig. 8). The average cytosine methylation levels of the Ri14 and Ri19 transgenic lines were 30.7% and 31.7%, respectively, which was significantly higher than that of the control at 19.9%, confirming that *JMJ524* plays a role in regulating DNA demethylation.

## Discussion

### JmjC domain-containing proteins play diverse functions in plants

Plant JmjC domain-containing proteins have important functions in epigenetic processes, gene expression, and plant development, as well as in the interplay between histone modification and DNA methylation. More than 20 JmjC domain-encoding genes have been found in *Arabidopsis* and rice, and these all showed higher conservation at the amino acid level than homologues from animals (Chen *et al.*, 2011*b*). Similar to other JmjC domain-containing proteins, *JMJ524* is highly conserved in different species (Fig. 1A), and a phylogenetic tree revealed that JMJ524 was closely related to JMJ30 and JMJD5 from *Arabidopsis* and human, respectively (Fig. 1B). The deduced protein sequence of JMJ524 shares 63% similarity with AtJMJ30 but only 35% similarity with HsJMJD5(data not shown). Consistent with its counterparts AtJMJ30, AtJMJD5, and HsJMJD5 (Jones and Harmer, 2011; Lu *et al.*, 2011), JMJ524 is also involved in a circadian clock response in tomato (Fig. 3B). However, knockdown of *JMJ524* causes a severe dwarfism phenotype in tomato, which is different from that of *Atjmj30* and *Atjmjd5* mutants (Jones and Harmer, 2011; Lu *et al.*, 2011), but similar to that of the *Arabidopsis ibm1* mutation (Saze *et al.*, 2008). Although no substantial similarity was found between IBM1 and JMJ524 at the amino acid sequence level, we still investigated whether *JMJ524* suppression would affect cell cycle regulators, in the same way as IBM1. However, unlike the *ibm1* mutation, which resulted in the epigenetic silencing of a gene encoding a homologue of a cell cycle regulator, BNS (Saze *et al.*, 2008), the *JMJ524-*RNAi lines showed increased expression of several cell cycle genes (Supplementary Table S3), consistent with the observation that the stem cell number of *JMJ524*-RNAi lines was significantly larger than that of the WT (Fig. 4E). In summary, these data suggest that JMJ524 and IBM1 might influence plant architecture through diverse mechanisms or species-specific differences.

### JMJ524 functions in the regulation of a GA response

Limited growth has a number of advantages for plants, such as preventing damage in cereal crops, curbing unwanted vegetative growth, improving the ratio of vegetative growth to fruit production, or reducing the size of ornamentals (Rademacher, 2000). Plant growth is driven by cell proliferation and elongation (Beemster and Baskin, 1998), and it has been confirmed that GAs regulate *Arabidopsis* root growth by controlling cell elongation and promoting root cell production (Ubeda-Tomas *et al.*, 2009). Plant growth retardants (PGRs) are used in agriculture to alter plant morphology by reducing shoot growth via a lowered rate of cell division and a reduction in cell elongation. Most PGRs that are used in agriculture or horticulture act in an antagonistic manner to GA and the phytohormone auxin (Ubeda-Tomas *et al.*, 2009). The *JMJ524-*RNAi plants showed a reduction in cell elongation (Fig.4D) that correlates with abnormal GA metabolism.

The phytohormone GA not only has a prominent role in the regulation of various developmental processes (Dill *et al.*, 2004), but is affected by the circadian clock, which gates GA signalling through transcriptional regulation of the GA receptors (Arana *et al.*, 2011). Here, we report on the response of *JMJ524* to the circadian rhythm. This was induced by GA_3_ treatment but inhibited in the *pro* mutant (Figs 3B, C and 5C). Moreover, the GA content increased, and GA biosynthetic genes were upregulated, in the *JMJ524-*RNAi transgenic lines (Fig. 5B). These data may suggest that *JMJ524* is involved in circadian oscillation of GA signalling. However, the function of *JMJ524* in GA biosynthesis contradicts the dwarf phenotype in the *JMJ524*-RNAi transgenic lines. Previous studies have shown that accumulation of DELLA protein would increase the expression levels of GA20ox and GA3ox genes, whereas downregulation of such protein following GA treatment would reduce the expression levels of these genes (Silverstone *et al.*, 2001; Itoh *et al.*, 2002). Based on these data, we hypothesized that the silenced *JMJ524* would upregulate the *DELLA* gene, leading to the dwarf phenotype. Upregulation of the *DELLA* gene would trigger improved expression of GA biosynthesis genes, such as GA20oxs and GA3oxs, through an unknown feedback mechanism. Moreover, the expression levels of GA2oxs do not show a consistent trend (some genes are upregulated and some are downregulated) (Table 1); how GA2oxs are regulated by DELLAs or JMJ524 is still unknown.

DELLAs are nuclear transcriptional regulators that repress GA signalling and restrict plant growth (Harberd *et al.*, 2009). The DELLA motif is essential for gibberellin-induced degradation of the DELLA protein (Dill *et al.*, 2001) and for allowing GA responses to occur (Harberd *et al.*, 2009). Deletions of the DELLA region in DELLA proteins would confer semi-dominant dwarf phenotypes in crops (Dill *et al.*, 2001), thereby reducing plant tolerance to cold and salt stresses (Sun, 2011). In the current study, two putative DELLA proteins lacking DELLA domains, namely *SlGLD1* and *SlGLD2*, were identified (Fig. 6A). *SlGLD1* and *SlGLD2* shared high similarities with the tomato DELLA protein Procera at the amino acid level (Bassel *et al.*, 2004) (Fig. 6B). All these DELLA homologues lack introns. *SlGLD1* overexpression in tomato resulted in a dwarf phenotype and induced the expression of GA biosynthesis and response (Fig. 7), suggesting that *SlGLD1* acts as a repressor of GA signalling. Given that *SlGLD1* does not contain the DELLA domain, which is essential for degradation of DELLA proteins, the resulting dwarfism of the tomato plants could not be recovered by exogenous GA_3_ application. However, *SlGLD2* overexpression showed no dwarf phenotype, which was consistent with the studies on two DELLA-like proteins from rice (SLRL1 and SLRL2) that have high sequence homology but lack DELLA domains. *SLRL1* overexpression in rice also induced a dwarf phenotype, whereas *SLRL2* was not involved in GA signalling (Itoh *et al.*, 2005). *SlGLD1* also induced a dwarf phenotype that was less severe than that of the *JMJ524-*RNAi lines. However, both plants displayed GA insensitivity, suggesting that *SlGLD1* is not the only target gene of *JMJ524* regulation throughout plant development.

### Knockdown of *JMJ524* confers altered GA responses via transcriptional regulation of *SlGLD1* by influencing DNA methylation

Recent research had shown that the JmjC-domain protein IBM1 controls genic DNA methylation in *Arabidopsis* (Saze *et al.*, 2008), that the *ibm1* mutation resulted in dwarf phenotypes, and that a rice histone lysine demethylase JMJ703 was also required for stem elongation (Chen *et al.*, 2013). These reports are consistent with our observation that *JMJ524* is required for stem elongation (Fig. 4), and induces the global DNA methylation level when it is inhibited (Fig. 8).

*JMJ524* suppression could confer altered gibberellin responses via transcriptional regulation of *SlGLD1.* However, how *JMJ524* regulates *SlGLD1* expression remains unknown. Knockout of *JMJ524* would cause a significant global increase in DNA methylation levels and inactivated genes may directly or indirectly regulate the expression of *SlGLD1* and other genes. *JMJ524* might also affect histone modifications, and less condensed chromatin remodelling could result in induction of the expression of *SlGLD1* and some other genes (Supplementary File S2).

At least 54 genes encoded a homologous amino acid sequence to the GRAS domain in the tomato genome (Jin *et al.*, 2014). Only a few GRAS proteins have been characterized to date. However, recent studies showed that GRAS proteins have important roles in plant development (Bolle, 2004) and environmental signals (Ma *et al.*, 2010). Nevertheless, the mechanisms of *JMJ524* and GRAS proteins in integrating environmental signals (such as the circadian rhythm) to restrict plant internode elongation remains unclear. Further studies on the relationship of *JMJ524* and GA-related GRAS proteins could provide a better understanding of how genes alter the GA response by integrating environmental signals or by DNA methylation to restrict plant development.

## Supplementary material

Supplementary data can be found at *JXB* online.

Supplementary Table S1. Primer sequences used for qPCR and gene cloning.

Supplementary Table S2. Total numbers of sequencing reads and sequencing quality analysis.

Supplementary Table S3. Differential expression of cyclin genes by RNA-seq analysis between *JMJ524*-RNAi (Ri) and WTs.

Supplementary Fig. S1. RNAi knockdown of *JMJ524* restricts tomato internode elongation.

Supplementary File S1. RNA-seq analysis showing differentially expressed genes comparing *JMJ524*-RNAi (Ri) and WT.

Supplementary File S2. Differential expression of methyltransferase and histone genes by RNA-seq analysis between *JMJ524*-RNAi (Ri) and WT.

Supplementary List S1. The GenBank accession or SGN numbers of JmjC proteins form *Arabidopsis thaliana* (At), *Oryza sativa* (Os), *Homo sapiens* (Hs), and tomato used for phylogenetic analysis in this research.

Supplementary List S2. The GenBank accession numbers of GRAS proteins used for phylogenetic analysis in this research.

## Funding

This work was supported by grants from the 863 Plan (2012AA100104), the National Science Foundation of China (31230064, 31171960, and 31301779), and the CARS-25-A-02.

## Supplementary Material

Supplementary Data
