# Supplementary material for: Knockdown of a JmjC domain-containing gene JMJ524 confers altered gibberellin responses by transcriptional regulation of GRAS protein lacking the DELLA domain genes in tomato
Source: J Exp Bot. 2015 Feb 13;66(5):1413–26. doi: 10.1093/jxb/eru493 (PMC4339600; doi:10.1093/jxb/eru493)
Supplement: Supplementary Data [file supp_66_5_1413__index.html]

Knockdown of a JmjC domain-containing gene JMJ524 confers altered gibberellin responses by transcriptional regulation of GRAS protein lacking the DELLA domain genes in tomato — Knockdown of a JmjC domain-containing gene JMJ524 confers altered gibberellin responses by transcriptional regulation of GRAS protein lacking the DELLA domain genes in tomato — Supplementary Data 

# Knockdown of a JmjC domain-containing gene *JMJ524* confers altered gibberellin responses by transcriptional regulation of GRAS protein lacking the DELLA domain genes in tomato

## Supplementary Data

Data files

**Files in this Data Supplement:**

- Supplementary Data - Supplementary Data
- Supplementary Data - Supplementary Data
- Supplementary Data - Supplementary Data
