# Supplementary material for: Knockdown of a JmjC domain-containing gene JMJ524 confers altered gibberellin responses by transcriptional regulation of GRAS protein lacking the DELLA domain genes in tomato
Source: J Exp Bot. 2015 Feb 13;66(5):1413–26. doi: 10.1093/jxb/eru493 (PMC4339600; doi:10.1093/jxb/eru493)
Supplement: Supplementary Data [file supp_eru493_jexbot135228_file003.pdf]

Knockdown of a JmjC domain-containing gene JMJ524 confers altered gibberellin responses by transcriptional regulation of GRAS protein lacking the DELLA domain genes in tomato. *Jinhua Li, Chuying Yu, Hua Wu, Zhidan Luo, Bo Ouyang, Long Cui, Junhong Zhang, and Zhibiao Ye*

SUPPLEMENTARY DATA

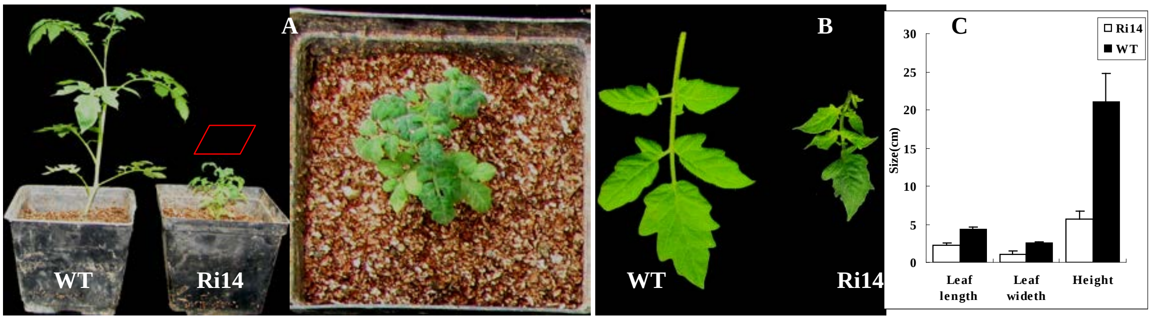

Supplementary Figure S1. RNAi knockdown *JMJ524* restricts the elongation of internode in tomato.

A. Phenotypic appearance of about 50 days old seedlings (left) of *JMJ524* RNAi-knockdown lines (Ri14) and wild-type (WT) plants, and right magnified view representing the plant with the red box.

B. The corresponding leaflet size of 50 days old WT and Ri14 seedlings.

C. The plant height and the third leaf from the top of 50 days old WT and Ri14 seedlings was used for leaflet size measurement. Error bar indicates SE (n=6).

Supplementary Table S1. **Primer sequences used for qPCR and gene cloning**

Primer		Sequence (5'-3')
β-Actin	qRT-PCR	Forward GTCCTCTTCCAGCCATCCAT<br>Reverse ACCACTGAGCACAATGTTACCG
GA3ox	qRT-PCR	Forward CTTACCACGCACTGGGGTTAGC<br>Reverse CAATGAGGGCATCGTTGGAAA
GA20ox 3	qRT-PCR	Forward AGCCAAATTATGCTAGTGTTAC<br>Reverse TTTTATGAGATTTGTGTCAACC
GA2ox 1	qRT-PCR	Forward CTCATTTCTAATGCTCATCGT<br>Reverse TGCAGATGATTCTTTCTTAGCG
GA2ox 2	qRT-PCR	Forward TTTCCATATTCTACCCTACAAG<br>Reverse TCATCGCATTACAATACTCTT
SlGID1	qRT-PCR	Forward AGAAACCTCTGAGCACCACG<br>Reverse ATCTTAACTAGGCGGCGACA
SlDELLA	qRT-PCR	Forward TGATGCGACTATACTTGATATAAG<br>Reverse GGGTTAATCTGTTTAATAGAGTTC
SlGLD1	qRT-PCR	Forward GGATCAAGTCTTGAGTATGAGCT<br>Reverse CATGTACTCTCTTGGAATCTGC
SlGLD2	qRT-PCR	Forward ATCCTATAACCATACGAGTGTCTACG<br>Reverse CAGGACGCAAAGCAAGAGC
JMJ524	qRT-PCR	Forward GGAGAAGGAGAAAGCGAAGA<br>Reverse AGGAATGCCTCCAACGATAA
JMJ524-OE	Overexpression vector	Forward AGAACACAACTGCATATCCACTATG<br>Reverse TGCCACTCTTTGGAAATTCA
JMJ524-Ri	RNAi vector	Forward (attB1) GGGGACAAGTTTGTACAAAAAAGCAGGCT-CTCTGCATCACTTTCGGATG<br>Reverse (attB2) GGGGACCACTTTGTACAAGAAAGCTGGGT-TGCCACTCTTTGGAAATTCA
CaMV35s		Forward ACGCACAATCCCACTATCCTTC
SlGLD1 OE	Overexpression vector	Forward TCCATATCCAAATGGGACCT<br>Reverse TTTGATTGCACTTGTCCTGC
SlGLD2 OE	Overexpression vector	Forward CGCGTCTTCCGTTGTTATTT<br>Reverse GCAGCAAGCTATTCCAGGAC
JMJ524-Y	Transcriptional activation in yeast	Forward CCCGGGCTGGATCCACATGTCCACGTCACCAGCCGA<br>Reverse CTGCAGGTCGACTCAGTCGTCTAATTTTTCAGCATCA

Supplementary Table S2 Total numbers of sequencing reads and sequencing quality analysis

Map to Gene	WT (wild-types)		Ri-14 (JMJ524-RNAi)	
	Reads number	Percentage (%)	Reads number	Percentage (%)
Total Reads	12 503 639	100.00%	11898385	100.00%
Total BasePairs	612678311	100.00%	583020865	100.00%
Total Mapped Reads	11137750	89.08%	10416898	87.55%
perfect match	9911938	79.27%	9263574	77.86%
<=2bp mismatch	1225812	9.80%	1153324	9.69%
unique match	10332756	82.64%	9843552	82.73%
multi-position match	804994	6.44%	573346	4.82%
Total Unmapped Reads	1365889	10.92%	1481487	12.45%

Supplementary Table S3. Differential expression of cyclin genes by RNA-seq analysis between JMJ524-RNAi (Ri) and wild-types (WT)

Gene ID	Gene length	log2 Ratio (Ri-14/WT)	Up or Down (Ri-14/WT)	P-value	Blast nr
Solyc10g074720.1.1	912	2.185451	Up	7.87E-06	B1-type cyclin
Solyc12g088530.1.1	1065	2.052696	Up	2.85E-09	cyclin A-like protein
Solyc05g051410.2.1	1228	1.558721	Up	3.95E-08	cyclin D2.1 protein
Solyc02g092980.2.1	1544	1.445014	Up	0	cyclin D3.1

List S1. HsJMJD5 (Q8N371), HsJMJD4 (Q9H9V9), AtJMJ20 (Q67ZB6), AtJMJ32 (Q9LZU2), OsJMJ712 (A3C049), OsJMJ717 (Q6YVS8), Solyc03g112600.2.1 , Solyc09g065690.2.1 , Solyc10g081630.1.1, Solyc08g075510.2.1, HsHSPBAP1 (Q96EW2), OsJMJ713 (Q5ZC07), AtJMJ30 (Q8RWR1), OsJMJ711 (Q75LR4), AtJMJ21 (Q9M9E8), HsKDM3A/JHDM2A (Q9Y4C1), OsJMJ716 (Q0DRY1), AtJMJ24 (Q27GN5), AtIBM1/JMJ25 (Q9SSE9), HsKDM4D/JHDM3D (Q6B0I6), AtREF6/JMJ12 (Q9STM3), AtELF6/JMJ11 (Q6BDA0) , AtJMJ13 (Q9FJS0), OsJMJ706 (Q8W3G5), HsKDM5B/JARID(Q9UGL1), AtMEE27/JMJ15 (O64752), AtJMJ14 (Q8GUI6), OsJMJ703 (Q53WJ1), HsKDM6B/JMJD3 (O15054), HsKDM6A/UTX (O15550)

List S2. *Arabidopsis thaliana*: AtRGA(CAA72177), At GAI (CAA75492), At RGL1 (AAL05911), At RGL2 (NP_186995), At RGL3 (NP_197251), At SCR (AAB06318) and At SCL28 (AAG51600); *Oryza sativa*: OsGRAS32 (BAA90816), Os SCR (BAD22576), Os SLR1 (BAE96289), Os SLRL1 (AAR31213) and Os SLRL2 (AAT69589); *Brassica rapamaize*: Br RGA1 (AAX33297) and Br RGA2 (AAX33298); *Cucumis sativus*: Cs SCR (CAI30892); *Hordeum vulgare*: Hv SLN1 (AAL66734); *Lupinus albus*: La SCR1 (ACQ84011) and La SCR2 (ACR48080); *Lactuca sativa*: Ls DELLA1 (BAG71200) and Ls DELLA2 (BAG71201); *Ipomoea nil*: Pn SCR (BAE48702); *Physcomitrella patens*: Pp DELLAL1 (ABX10764) and Pp DELLAL2 (ABX10765); *Pinus sylvestris*: Ps SCR (ABH85406); *Populus trichocarpa*: PtGRAS18 (EEE84364) and PtGRAS19 (EEE78544); Ricinus communis: Rc GAI1 (EEF49399) and Rc RGL1 (EEF51101); *Triticum aestivum*: TaRHT-D1A (CAB51555); *Selaginella moellendorffii*: Sm DELLA1 (ABX10758); *Vitis vinifera*: Vv GAI1 (AAM19210); Zea mays: Zm DWARF8 (CAB51557), Zm DWARF9 (ABI84226) and Zm SCR (AAG13663)
